# For-profit hospitals have a unique opportunity to serve as anchor institutions in the U.S

**DOI:** 10.1016/j.pmedr.2021.101372

**Published:** 2021-04-03

**Authors:** Cory E. Cronin, Berkeley Franz, Kelly Choyke, Vanessa Rodriguez, Brian K. Gran

**Affiliations:** a Ohio University, Department of Social and Public Health, Grover W359, Athens, OH 45701, United States; b Ohio University, Heritage College of Osteopathic Medicine, Department of Social Medicine, Grosvenor 311, Athens, OH 45701, United States; c Case Western Reserve University, Department of Sociology, Mather Memorial Building 224, 10900 Euclid Avenue, Cleveland, OH 44106, United States

**Keywords:** Hospitals, For-profit, Anchor Institutions, Social Determinants of Health, Population Health

## Abstract

Hospitals serve as anchor institutions in many U.S. communities and make contributions to bolster population health and reduce preventable death. Most studies to date have focused on nonprofit hospitals, but there may be significant opportunity for for-profits to fill this role in both urban and rural communities. Using 2017–2018 data, we calculated descriptive statistics and a multivariate regression model to assess economic and health characteristics for all U.S. counties that contain for-profit as compared to nonprofit or public hospitals (n = 4,622). After controlling for hospital and county characteristics, we found a significant and positive relationship between for-profit hospital presence and higher county unemployment, higher uninsured rates, and the number of residents reporting poor/fair health. For-profit hospitals were also less likely to be located in states that had expanded Medicaid or which had certificate-of-need laws. Our findings suggest that there is substantial opportunity for for-profit hospitals to serve as anchor institutions in many U.S. communities, despite this label more traditionally being applied to nonprofit hospitals. Given that there is not currently a regular reporting mechanism for documenting the community health contributions of for-profit hospitals, policymakers and researchers should evaluate the current state of these contributions and develop incentives to encourage more anchor activities to benefit economically vulnerable communities in the U.S.

## Highlights

• For-profit hospitals are more likely to operate in vulnerable communities.
• For-profit hospitals have potential to serve as anchor institutions.
• Policymakers should identify new incentives for hospital community engagement.
• Incentives must extend beyond community benefit standards for tax exempt hospitals.

## 1 Introduction

Hospitals serve as prominent public health partners in U.S. communities and make contributions to bolster population health and improve economic conditions. These investments are vital to addressing health disparities and reducing preventable deaths (Mays et al., 2016). Although nonprofit hospitals are legally required to undertake population health activities, no data are available regarding the population health investments of for-profit or public hospitals. Given recently implemented and proposed reimbursement mechanisms, incentives have the potential to encourage for-profit hospitals to invest in improving health in their surrounding communities (Garg et al., 2019, Houlihan and Leffler, 2019). It is not clear, however, what the economic and public health impacts would be if for-profit hospitals directed more resources toward population health improvement.

The vast majority of hospitals currently operating in the United States are in the corporate form, meaning they are either privately owned nonprofit or for-profit hospitals. While government-funded public hospitals still comprise approximately 18.5% of the U.S. market, today nonprofits and for-profits control 81.5% of the American hospital market (American Hospital Association, 2020). Although more than half of hospitals nationwide are nonprofit, for-profit hospitals are growing in number and compose approximately a quarter of hospitals (American Hospital Association, 2020).

Hospitals have long been considered central to the welfare of a community, given their mission to provide acute medical services. However, hospitals also fill important nonmedical roles in both urban and rural communities (Rozier et al., 2019, Corrigan et al., 2015, Cronin, 2017). For example, hospitals frequently are the largest employers in their communities and are less likely than other businesses to leave the community and relocate elsewhere. For this reason, hospitals are often referred to as anchor institutions because they have strong potential to bolster economic development within communities and increase well-being (Zuckerman et al., 2013, Norris and Howard, 2015).

An ‘anchor institution’ is a large institution – typically an educational, health, or other large corporation – that is deeply embedded within the economic infrastructure of a community, and also plays a role in improving community life. In the case of hospitals, this may include community health (Franz et al., 2018, Corbett and Kappagoda, 2013, Koh et al., 2020, John and de Socio, 2014, Skinner et al., 2016, Louis and Luter, 2013). Beyond job opportunities, anchor institutions invest in the social, economic, and health development of local communities as a significant part of their business models (Louis and Luter, 2013, Devereaux et al., 2002). Koh and colleagues describe anchors as institutions that “commit major financial, human, and intellectual resources to address social challenges, understanding that their future is inextricably linked to the community outside their walls” (10 p. 309). While hospitals in general have been discussed as having the potential to be anchor institutions (Louis and Luter, 2013, Devereaux et al., 2002), only nonprofits have been typically described as such (Franz et al., 2018). The result is that very little literature exists on the public health potential of for-profit institutions.

For-profit hospitals do have documented differences in comparison to nonprofits, and these differences may affect the potential for for-profit organizations to function as anchor institutions. For example, for-profit hospitals are more efficient in terms of employees, which may limit the numbers of jobs they provide to the local community (Hirth, 1997). For-profits are also more likely to offer services that are profitable, as opposed to unprofitable but necessary, which may impact access to critical health care services in medically underserved settings (Horwitz, 2005, Horwitz and Nichols, 2009, Horwitz and Nichols, 2011, Shortell et al., 1986, Bolon, 2005).

Some scholars have argued that there are no significant differences in mission across for-profit and nonprofit hospitals and have questioned why most discussion of hospital anchors has focused on nonprofits in particular (Meier et al., 2019, Cram et al., 2010). There do not seem to be significant differences in the amount of uncompensated care offered by each hospital type (Duggan, 2000, Hultman, 1991, Norton and Staiger, 1994, Clement et al., 2002, Valdovinos et al., 2015, McCue, 2007), and there is evidence to suggest that for-profits serve a larger proportion of Medicaid patients, especially in rural markets (David, 2009).

Less is known about hospital decisions to enter health care markets and the distribution of for-profit vs. nonprofit hospitals in communities where institutional investments would have significant potential to elevate local population health. Regulatory and market changes, such as the introduction of the Medicare and Medicaid Prospective Payment System (PPS) in 1983, and ensuing changes to state and federal policy governing nonprofits and for-profits on a state by state basis, have created a mixed medical market in the U.S. that varies by state and region, with some states having more desirable markets for for-profits, such as Texas and Florida, and some states that do not have desirable, or open markets, for for-profits, such as New York, Maryland, and Vermont (Shortell et al., 1986, Nicholson et al., 2000, Shortell and Hughes, 1988, Chiu et al., 2019, Koopman and Philpot, 2016). There is evidence that for-profit hospitals may choose to locate in communities where there is less competition and better reimbursement rates (Kaiser Family Foundation, 2020) but more research is needed to understand the regional distribution of for-profit hospitals and the social and economic characteristics of the communities in which they are located.

The aim of this study, accordingly, is to understand the potential impact of for-profit hospitals serving in an anchor role, particularly if these organizations were willing to undertake new investments in community health. In other words, we explore if for-profit hospitals are located in unique social and economic environments as compared to their nonprofit and public hospital counterparts. This manuscript contributes to the existing literature in two key ways. First, the results will identify factors that increase the likelihood of hospitals of different ownership types operating in a county. Second, these results will provide insight into economic and health needs in the counties where different types of hospitals are located and will, as a result, illuminate the opportunity for health improvement if for profit hospitals were to engage in population health initiatives.

## 2 Methods

Our data come from the United States Census American Community Survey, the Area Health Resource File and the American Hospital Association (AHA) Annual Survey. Community characteristics from all data sources are from the year 2017, while hospital characteristics are from 2018 data. The dataset also includes data on 2016 state certificate-of-need laws from the Mercatus Center at George Mason University (Mislinski, 2019) state Medicaid expansion data from the Kaiser Family Foundation (Lee et al., 2017) and purchasing power data sourced from the U.S. Census Bureau Current Population Survey and the Council for Community and Economic Research, congregated by advisorperspectives.com (Paul et al., 2020).

### 2.1 Sample

This study assesses characteristics as they relate to a hospital’s presence in a county. The sample for the descriptive portion of the study consists of all U.S. hospitals and the counties within which they are located. The sample for the analytic portion of the study consists of U.S. counties with general medical hospitals. This definition is distinct from AHA’s community hospital definition, in that it excludes hospitals that specialize (e.g. orthopedic or ear, nose and throat hospitals) since these tend to be smaller facilities that may not take part in a full-range of anchor related activities. The sample also excludes federal hospitals but includes other public hospitals. Counties with multiple general medical hospitals are observed multiple times in the sample. After accounting for missing data, the analytic sample for this study is 4,622 county observations. Eighteen hospitals were dropped because of missing county-level information, and one was dropped because of data missing on an AHA variable. For this reason, these observations were not included in the analysis.

### 2.2 Analytic strategy

In addition to the use of descriptive statistics, this study employs multivariate logistic regression. The key dependent variable for this analysis is the presence of a for-profit hospital within a county, considering county characteristics and hospital characteristics. We calculate the odds of a county containing a for-profit hospital in four individual models, each with a key county health or economic measure. Key measures include one health indicator, the percent of the population reporting poor or fair health, and three economic variables: unemployment, uninsured rate, and median per capita income. To offer a comparison between hospital ownership types, we further calculate the same models in relation to nonprofit hospital presence and then in relation to public hospital presence. The analysis also considers hospital characteristics (size, system membership, and teaching status), community and geographic characteristics (number of hospitals in county adjusted for population, percent of population white, and rural location), and policy characteristics (state has certificate-of-need regulation in place and status of Medicaid expansion) (measures described in Table 1).

**Table 1:** Descriptive statistics of county and hospital characteristics.

Variable:	Description and source:	Total Sample N = 4622		For-profit general medical hospitals N = 694		Nonprofit general medical hospitals N = 2,780		Public general medical hospital N = 957	
		Frequency	Percent	Frequency	Percent	Frequency	Percent	Frequency	Percent
Beds size:	Categories of total hospital beds; AHA 2018 Annual Survey								
Fewer than 50		1,613	34.91%	182	26.22%	827	29.75%	559	58.41%
Beds 50–199		1,655	35.81%	321	46.25%	1,009	36.29%	246	25.71%
Beds 200–399		874	18.91%	149	21.47%	599	21.55%	79	8.25%
Beds greater than 400		480	10.39%	42	6.05%	345	12.41%	73	7.63%
System member	Hospital is member of a health system; AHA 2018 Annual Survey	3,096	66.98%	586	84.44%	2,041	73.42%	284	29.68%
Teaching status	Hospital is a major teaching hospital; AHA 2018 Annual Survey	286	6.19%	6	0.86%	196	7.05%	50	5.22%
Rural location	County is non-metro on the USDA ERS classification; sourced Area Health Resource File	1,872	40.50%	178	25.65%	1,016	36.55%	639	66.77%
State has CON regulation	State has certificate-of-need law in place; https://www.mercatus.org	2,892	62.57%	408	58.79%	1,760	63.31%	594	62.07%
State expansion of Medicaid	State has expanded Medicaid; khn.org	2,651	57.36%	262	37.75%	1,837	66.08%	439	45.87%
Region:	U.S. region; Census Bureau								
Northeast		561	12.14%	35	5.04%	477	17.16%	28	2.93%
Midwest		1,379	29.84%	74	10.66%	969	34.86%	293	30.62%
West		952	20.60%	154	22.19%	522	18.78%	221	23.09%
South		1,730	37.43%	431	62.10%	812	29.21%	415	43.36%

**County Characteristics**		**N**	**Mean (SD)**	**N**	**Mean (SD)**	**N**	**Mean (SD)**	**N**	**Mean (SD)**

Percent unemployed	Percent of county residents unemployed; sourced Area Health Resource File from U.S. Census	4,622	5.5 (1.42)	694	4.59 (1.23)	2,780	4.44 (1.34)	957	4.54 (1.71)
Percent uninsured	Percent of county residents under age 65 without health insurance; sourced Area Health Resource File from U.S. Census	4,622	10.68 (4.93)	694	13.19 (5.32)	2,780	9.49 (4.3)	957	12.28 (12.28)
Percent reporting poor/fair health	Percent of county residents reporting health status as fair or poor; County Health Rankings	4,622	16.83 (4.14)	694	18.35 (4.19)	2,780	16.13 (3.81)	957	17.6 (4.49) %
Percent population white	Percent of the population single race, white non-Hispanic; Area Health Resource File	4,622	77.15 (19.76)	694	72.80 (18.97)	2,780	79.13 (19.31)	957	75.67 (19.98)
Adjusted median income	County median income adjusted for purchasing power*	4,622	$56172 ($15438)	694	$54948 ($14442)	2,780	$58228 ($16008)	957	$51300 ($13261)
Hospitals adjusted for population	Number of hospitals in a county per 1,000 residents; Area Health Resource File	4,622	0.46 (0.78)	694	0.24 (0.33)	2,780	0.37 (0.64)	957	0.93 (1.18)

## 3 Results

**Fig. 1:**
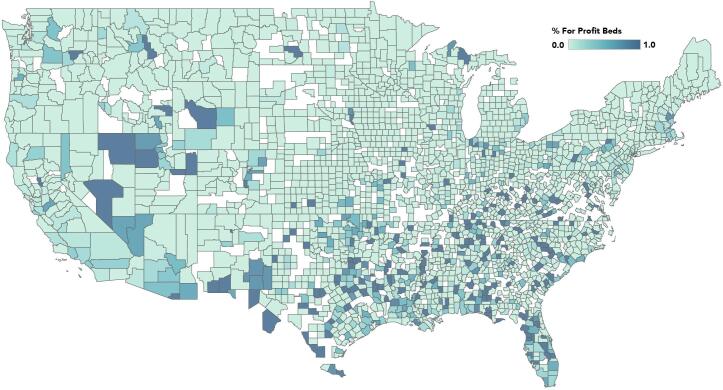
Geographic distribution of for-profit hospital beds in the US. Using the percentage of beds in a county that were in for-profit hospitals, this map indicates where for profit hospitals are most likely to operate. Darker colors on the map indicate a higher percentage of for-profit beds.

Descriptive statistics identified 694 for-profit general medical centers, which account for 15% of the 4622 hospitals in the analytic sample. We identified 243 counties in the U.S. as having only for-profit hospitals, whereas 1,373 counties across the country have at least one non-profit hospital and no for-profit hospitals. The majority of counties with for-profit hospitals are also served by non-profit or public hospital facilities. As Fig. 1 shows, for-profit hospitals are not consistently distributed across the country and are instead concentrated in certain regions.

**Fig. 2:**
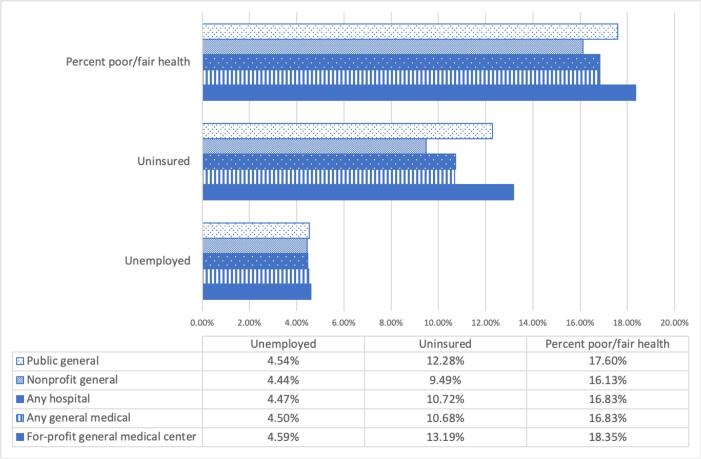
County characteristics associated with hospital type. Using descriptive statistics, this figure shows whether different types of hospitals are located in counties with significant health and economic needs. For profit general medical centers are located in counties with higher unemployment, uninsurance, and poor/fair physical health than other hospital types.

**Fig. 3:**
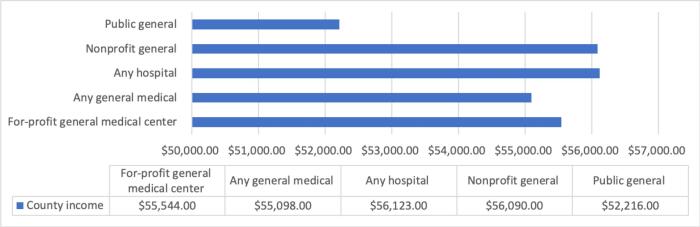
County income associated with hospital type. Using descriptive statistics, this figure shows the average county income (adjusted for purchasing power) where different types of hospitals are located. Counties with public hospitals have the lowest average income; counties with for profit hospitals have lower average income than counties with nonprofit hospitals.

The counties assessed in this study average a 5.5% unemployment rate; a 10.7% uninsured rate; and a 16.8% rate of reporting poor or fair health, with a median income (adjusted for purchasing power) of $56,000. However, counties with for-profit hospitals report higher averages in uninsurance rates (13%) and in reporting poor or fair health (18%) compared to nonprofits (9.5% and 16.1%, respectively) (Fig. 2). This gap between the means for the total sample and the for-profit hospitals’ counties widens when comparing nonprofit hospitals’ counties and for-profit hospitals’ counties. Descriptive statistics also indicated that counties with for-profit hospitals had, on average, a lower median income (adjusted for purchasing power) than those with nonprofit hospitals (Fig. 3).

Our multivariate analysis provides further understanding of the county characteristics associated with for-profit hospital presence (See Table 2). The analysis presented a significant relationship between for-profit hospital presence and higher county unemployment, higher uninsured rates, and the number of residents reporting poor/fair health. For-profit hospital presence was also significantly associated with lower median income (adjusted for purchasing power), though the effect for this variable was low.

**Table 2:** Multivariate results of characteristics associated with for-profit hospital presence.

*Continuous key variable N = 4,622 counties with general medical centers*												
	Unemployed			Uninsured			Poor/Fair Health			Purchasing power/Adjusted median income		
	OR (SE)	P	95% Conf Int	OR (SE)	P	95% Conf Int	OR (SE)	P	95% Conf Int	OR (SE)	P	95% Conf Int
key variable	1.12 (0.04)	0.001	[1.05–1.19]	1.1 (0.01)	0.000	[1.07–1.13]	1.1 (0.01)	0.000	[1.08–1.13]	0.99 (0)	0.002	[0.98–1]
Hospitals in market (adjusted for population)	0.46 (0.08)	0.000	[0.32–0.66]	0.43 (0.08)	0.000	[0.31–0.61]	0.44 (0.08)	0.000	[0.3–0.63]	0.44 (0.08)	0.000	[0.31–0.63]
County percent white	1 (0)	0.077	[1–1.01]	1.01 (0)	0.032	[1–1.01]	1 (0)	0.051	[1–1.01]	1 (0)	0.061	[1–1.01]
Rural location	0.59 (0.07)	0.000	[0.46–0.75]	0.55 (0.07)	0.000	[0.43–0.71]	0.49 (0.06)	0.000	[0.38–0.63]	0.56 (0.07)	0.000	[0.44–0.72]
Hospital part of system	2.52 (0.3)	0.000	[1.99–3.18]	2.63 (0.32)	0.000	[2.07–3.33]	2.62 (0.32)	0.000	[2.07–3.33]	2.45 (0.29)	0.000	[1.94–3.1]
Teaching hospital	0.14 (0.06)	0.000	[0.06–0.33]	0.14 (0.06)	0.000	[0.06–0.32]	0.14 (0.06)	0.000	[0.06–0.32]	0.14 (0.06)	0.000	[0.06–0.32]
Bed size: 50–199	1.11 (0.13)	0.356	[0.89–1.4]	1.18 (0.14)	0.157	[0.94–1.5]	1.09 (0.13)	0.447	[0.87–1.38]	1.1 (0.13)	0.409	[0.88–1.39]
Bed size: 200–399	0.75 (0.11)	0.051	[0.57–1]	0.76 (0.11)	0.058	[0.57–1.01]	0.72 (0.1)	0.023	[0.54–0.95]	0.74 (0.11)	0.037	[0.56–0.98]
Bed size: greater than 400	0.46 (0.1)	0.000	[0.3–0.69]	0.44 (0.09)	0.000	[0.29–0.67]	0.43 (0.09)	0.000	[0.28–0.65]	0.45 (0.09)	0.000	[0.3–0.67]
State has CON law	0.62 (0.06)	0.000	[0.5–0.76]	0.78 (0.08)	0.021	[0.64–0.96]	0.63 (0.07)	0.000	[0.51–0.77]	0.6 (0.06)	0.000	[0.49–0.74]
State has expanded Medicaid	0.52 (0.07)	0.000	[0.41–0.67]	0.87 (0.12)	0.314	[0.66–1.14]	0.49 (0.06)	0.000	[0.38–0.62]	0.53 (0.07)	0.000	[0.42–0.68]
Region: Northeast	0.26 (0.06)	0.000	[0.17–0.4]	0.36 (0.08)	0.000	[0.23–0.56]	0.41 (0.09)	0.000	[0.26–0.64]	0.25 (0.05)	0.000	[0.16–0.38]
Region: Midwest	0.22 (0.04)	0.000	[0.16–0.3]	0.33 (0.06)	0.000	[0.23–0.46]	0.32 (0.05)	0.000	[0.23–0.44]	0.22 (0.03)	0.000	[0.16–0.3]
Region: West	0.67 (0.1)	0.009	[0.5–0.9]	0.83 (0.13)	0.242	[0.61–1.13]	0.94 (0.15)	0.703	[0.69–1.29]	0.63 (0.1)	0.002	[0.47–0.85]

***Non-profit comparison (probably to include as appendix)***												

*Continuous key variable N = 4,622 counties with general medical centers*												

	**Unemployed**			**Uninsured**			**Poor/Fair Health**			**Purchasing power/Adjusted median income**		
	OR (SE)	P	95% Conf Int	OR (SE)	P	95% Conf Int	OR (SE)	P	95% Conf Int	OR (SE)	P	95% Conf Int

key variable	0.94 (0.02)	0.008	[0.89–0.98]	0.93 (0.01)	0.000	[0.92–0.95]	0.93 (0.01)	0.000	[0.92–0.95]	1.01 (0)	0.016	[1–1.01]
Hospitals in market (adjusted for population)	0.77 (0.04)	0.000	[0.69–0.85]	0.81 (0.04)	0.000	[0.73–0.9]	0.75 (0.04)	0.000	[0.68–0.84]	0.79 (0.04)	0.000	[0.71–0.87]
County percent white	1 (0)	0.506	[1–1]	1 (0)	0.746	[1–1]	1 (0)	0.672	[1–1]	1 (0)	0.526	[1–1]
Rural location	1.18 (0.1)	0.050	[1–1.4]	1.23 (0.11)	0.016	[1.04–1.45]	1.32 (0.12)	0.001	[1.12–1.57]	1.22 (0.11)	0.024	[1.03–1.46]
Hospital part of system	1.89 (0.14)	0.000	[1.64–2.17]	1.86 (0.13)	0.000	[1.62–2.15]	1.87 (0.13)	0.000	[1.62–2.15]	1.9 (0.14)	0.000	[1.65–2.19]
Teaching hospital	0.68 (0.11)	0.022	[0.49–0.95]	0.69 (0.11)	0.023	[0.5–0.95]	0.7 (0.12)	0.032	[0.51–0.97]	0.69 (0.11)	0.025	[0.5–0.96]
Bed size: 50–199	1.2 (0.1)	0.023	[1.03–1.41]	1.17 (0.1)	0.063	[0.99–1.37]	1.22 (0.1)	0.018	[1.03–1.43]	1.21 (0.1)	0.022	[1.03–1.42]
Bed size: 200–399	1.63 (0.18)	0.000	[1.32–2.02]	1.63 (0.18)	0.000	[1.32–2.02]	1.68 (0.18)	0.000	[1.35–2.08]	1.64 (0.18)	0.000	[1.33–2.03]
Bed size: greater than 400	2.23 (0.34)	0.000	[1.67–3]	2.29 (0.35)	0.000	[1.7–3.08]	2.3 (0.35)	0.000	[1.71–3.09]	2.26 (0.34)	0.000	[1.68–3.03]
State has CON law	1.05 (0.08)	0.546	[0.9–1.21]	0.94 (0.07)	0.420	[0.81–1.09]	1.05 (0.08)	0.519	[0.91–1.21]	1.04 (0.08)	0.568	[0.9–1.21]
State has expanded Medicaid	1.85 (0.16)	0.000	[1.57–2.19]	1.3 (0.12)	0.005	[1.08–1.57]	1.89 (0.16)	0.000	[1.6–2.23]	1.81 (0.15)	0.000	[1.54–2.14]
Region: Northeast	3.79 (0.56)	0.000	[2.83–5.08]	3.07 (0.47)	0.000	[2.27–4.13]	2.76 (0.43)	0.000	[2.03–3.74]	3.94 (0.58)	0.000	[2.94–5.26]
Region: Midwest	2.47 (0.24)	0.000	[2.04–3]	1.92 (0.21)	0.000	[1.56–2.37]	1.9 (0.2)	0.000	[1.55–2.34]	2.48 (0.25)	0.000	[2.05–3.01]
Region: West	1 (0.11)	0.966	[0.81–1.24]	0.92 (0.1)	0.427	[0.74–1.14]	0.79 (0.09)	0.035	[0.63–0.98]	1.03 (0.11)	0.765	[0.84–1.28]

***Public comparison (probably to include as appendix)***												

*Continuous key variable N = 4,622 counties with general medical centers*												

	**Unemployed**			**Uninsured**			**Poor/Fair Health**			**Purchasing power/Adjusted median income**		
	OR (SE)	P	95% Conf Int	OR (SE)	P	95% Conf Int	OR (SE)	P	95% Conf Int	OR (SE)	P	95% Conf Int

key variable	0.98 (0.03)	0.436	[0.92–1.04]	0.99 (0.01)	0.451	[0.97–1.02]	1 (0.01)	0.861	[0.98–1.03]	1 (0)	0.702	[0.99–1.01]
Hospitals in market (adjusted for population)	1.45 (0.08)	0.000	[1.29–1.62]	1.46 (0.08)	0.000	[1.31–1.64]	1.46 (0.08)	0.000	[1.3–1.63]	1.46 (0.08)	0.000	[1.3–1.64]
County percent white	1 (0)	0.274	[0.99–1]	1 (0)	0.276	[0.99–1]	1 (0)	0.316	[0.99–1]	1 (0)	0.290	[0.99–1]
Rural location	1.9 (0.21)	0.000	[1.53–2.35]	1.9 (0.21)	0.000	[1.53–2.35]	1.87 (0.21)	0.000	[1.5–2.32]	1.9 (0.22)	0.000	[1.52–2.38]
Hospital part of system	0.16 (0.01)	0.000	[0.14–0.19]	0.16 (0.01)	0.000	[0.14–0.19]	0.16 (0.01)	0.000	[0.14–0.19]	0.16 (0.01)	0.000	[0.14–0.19]
Teaching hospital	1.94 (0.43)	0.003	[1.26–3]	1.95 (0.43)	0.003	[1.26–3.01]	1.94 (0.43)	0.003	[1.26–3]	1.95 (0.43)	0.003	[1.26–3.01]
Bed size: 50–199	0.62 (0.06)	0.000	[0.5–0.75]	0.61 (0.06)	0.000	[0.5–0.75]	0.62 (0.06)	0.000	[0.5–0.75]	0.62 (0.06)	0.000	[0.5–0.75]
Bed size: 200–399	0.55 (0.09)	0.000	[0.41–0.75]	0.55 (0.09)	0.000	[0.41–0.75]	0.55 (0.09)	0.000	[0.41–0.75]	0.55 (0.09)	0.000	[0.41–0.75]
Bed size: greater than 400	0.9 (0.18)	0.616	[0.61–1.34]	0.91 (0.18)	0.629	[0.61–1.35]	0.91 (0.18)	0.627	[0.61–1.34]	0.91 (0.18)	0.627	[0.61–1.34]
State has CON law	1.26 (0.12)	0.019	[1.04–1.52]	1.23 (0.12)	0.037	[1.01–1.49]	1.24 (0.12)	0.025	[1.03–1.5]	1.25 (0.12)	0.022	[1.03–1.51]
State has expanded Medicaid	0.71 (0.08)	0.002	[0.58–0.88]	0.67 (0.08)	0.001	[0.53–0.85]	0.7 (0.07)	0.001	[0.57–0.86]	0.7 (0.07)	0.001	[0.57–0.87]
Region: Northeast	0.23 (0.05)	0.000	[0.15–0.37]	0.23 (0.05)	0.000	[0.14–0.36]	0.24 (0.06)	0.000	[0.15–0.39]	0.24 (0.05)	0.000	[0.15–0.37]
Region: Midwest	0.69 (0.09)	0.004	[0.54–0.89]	0.67 (0.09)	0.004	[0.52–0.88]	0.71 (0.1)	0.014	[0.55–0.93]	0.7 (0.09)	0.005	[0.54–0.9]
Region: West	1.17 (0.16)	0.250	[0.89–1.53]	1.16 (0.16)	0.287	[0.88–1.52]	1.19 (0.17)	0.242	[0.89–1.58]	1.18 (0.16)	0.236	[0.9–1.54]

For-profit hospitals have significantly lower odds of being in markets where there are more hospitals, of being in rural areas, or of being in regions outside the South. State regulations are also associated with for-profit hospital presence; for-profit hospitals have significantly lower odds of being in states with certificate-of-need laws or of being in states that have expanded Medicaid. Certain hospital characteristics were also significant, with for-profit hospitals having lower odds of being teaching hospitals or having 400 or more beds and higher odds of being a part of a multi-hospital system.

## 4 Discussion

The aim of this study was to assess whether for-profit hospitals are more likely than other hospitals to operate in counties with significant health and economic needs. Our findings provide insight into the geographic distribution of for-profit hospitals and suggest that for-profit hospitals could have a disproportionate impact on population health if they were successfully incentivized to engage in population health improvement. Although previous research suggested that for-profit hospitals often seek out counties where they can hold significant market share and provide profitable healthcare services (Horwitz and Nichols, 2009, Shortell et al., 1986, Sloan et al., 2001, McCue, 2007, GWU School of Business, 2020, Alexander et al., 1996), we find that the majority of for-profit hospitals in the United States are located in counties where nonprofit hospitals are also operating. We do find important regional differences, however, and observe lower for-profit presence in states that have certificate-of-need laws and that have expanded Medicaid.

At their core, both nonprofit and for-profit hospitals are privately held corporations, but much of the previous literature has focused on the potential contributions that nonprofits make to their local communities as a result of community benefit requirements in exchange for the tax exemption that these hospitals receive. For-profit hospitals, by contrast, pay taxes and may disperse their revenue streams to investors and leadership (Cuellar and Gertler, 2003). Because of this tax status, however, they may also make important contributions to the local tax base, particularly in communities that have experienced economic decline and the loss of major institutions. However, our findings suggest that for-profits are likely to have an even greater opportunity to directly impact population health based on their location in counties with significant economic and health needs. Though the data do not provide any indication as to why this relationship exists, it is possible that for-profit hospitals enter counties when other hospitals have failed, potentially due to poor economic and physical health which may make services less profitable (Joynt et al., 2014, Franz et al., 2019). Indeed, existing data suggest that when for-profit hospitals purchase nonprofit hospitals, the former hospitals were more likely to be small institutions in need of financial security brought on by a for-profit hospital system (Bazzoli et al., 2010).

Regardless of the mechanism by which for-profit hospitals enter counties with significant economic and health needs, for-profit hospitals appear to have the potential to serve as economic anchors in some of the same ways their nonprofit counterparts do: by providing jobs to local residents, incentivizing their employees to live nearby and support the local economy, and through the acquisition of hospital supplies from local businesses (Young et al., 2013). This does not mean that for-profits are currently doing this work, but these institutions should be considered as possible partners in population health improvement initiatives. Much of this work may be passive in nature, such as with employment or supplier diversity programs, and may occur because it is considered sound business practice or as an effort to improve employee health and well-being. However, if for-profit hospitals are able to recognize the role they play within their communities and their ability to affect the community’s well-being, economic or otherwise, these institutions may find it mutually beneficial to themselves and their communities to leverage this potential by building stronger cross-sector partnerships. Future research should utilize qualitative methods to understand the prioritization of population health initiatives within for-profit hospitals and whether specific incentives would increase the adoption of these activities.

Although data are not currently available to ascertain the specific investments that for-profit hospitals undertake to improve local population health, our findings underscore the potential for for-profit hospitals to greatly benefit the communities in which they are located and the need for better data on population health investments. Following previous research on community health investments, the tax status of hospitals may not be the only or most important factor in assessing their potential contributions to population health (Horwitz and Nichols, 2009, Horwitz and Nichols, 2011, Nicholson et al., 2000). In fact, a body of evidence suggests that on average hospitals, do not invest significantly in community health improvement (Rosenbaum, 2016, Ginn and Moseley, 2006, Herring et al., 2018). Most charitable activities, instead, are focused on charity care or on Medicaid shortfalls. Given that the effectiveness of community benefit laws for nonprofit hospitals remain disputed (Shortell and Hughes, 1988) policymakers might consider alternative mechanisms to both report the contributions that for-profit hospitals make to their surrounding communities (53) and incentivize anchor activities among all hospitals to reduce health disparities, especially in urban areas where for-profit hospitals are most likely to be located.

## 5 Limitations

Our findings may be limited by the use of counties as a proxy for the community served by a hospital. While county statistics do provide context for the environment within which a hospital operates, a hospital’s primary service area may not reach the full county or may extend beyond it limiting our ability to understand what impact for-profit hospitals may have on the broader community. Because our data are cross-sectional, we cannot assess whether the presence of a for-profit hospital contributes to poor economic or health outcomes or whether for-profit hospitals may identify markets with these characteristics as better suited for their organizations. Additionally, longevity and stability are important factors for anchor institutions. This study does not examine that aspect of the for-profit hospital sector, but future research on this topic should take this into consideration.

## 6 Conclusion

For-profit hospitals are disproportionately likely to be located in counties with significant economic and physical health needs. As such, there is substantial opportunity for for-profit hospitals to serve as anchor institutions in many U.S. communities, despite this label more traditionally being applied to nonprofit hospitals. Although for-profit hospitals do not have the same federal tax requirements to contribute to community health and well-being, there are significant financial incentives to contribute to population health improvement. Given that there is not currently a regular reporting mechanism for documenting the community health contributions of for-profit hospitals, policymakers and researchers should evaluate the current state of these contributions and identify incentives to encourage more anchor activities to benefit economically vulnerable communities in the U.S.

## 7 Availability of data and materials

The datasets generated and analyzed in this study are available from the following publicly available sources:

• United States Census American Community Survey (2017): https://data.census.gov/cedsci/
• Area Health Resource File (2017): https://data.hrsa.gov/data/download
• Mercatus Center: https://www.mercatus.org/publications/corporate-welfare/state-certificate-need-laws-2016
• Kaiser Family Foundation: https://www.kff.org/medicaid/issue-brief/status-of-state-medicaid-expansion-decisions-interactive-map/
• US Census Bureau Current Population Survey and Council for Community and Economic Research, congregated by advisorperspectives.com: https://www.advisorperspectives.com/dshort/updates/2019/12/19/median-household-purchasing-power-for-the-50-states-and-dc

The current study also generated and analyzed data from the American Hospital Association Annual Survey (2018), which are available for purchase from https://www.aha.org/data-insights/aha-data-products.

## 8 Declarations

*Ethics approval and consent to participate:* Not applicable.

*Consent to publish:* Not applicable.

*Competing interests:* The authors declare that they have no competing interests.

*Funding:* This study was supported by the Robert Wood Johnson Foundation. This funding supported access to the American Hospital Association Annual Survey, the primary source of data for this study.

*Authors’ contributions:* CEC, BF, and BG contributed to the conceptualization of the study. CEC and VR organized and managed data. CEC performed the statistical analysis. CEC, BF, KC wrote the initial draft of the manuscript. All authors contributed to manuscript writing and editing and approved the final draft of the manuscript.

## Declaration of Competing Interest

The authors declare that they have no known competing financial interests or personal relationships that could have appeared to influence the work reported in this paper.
